# Plumage quality mediates a life-history trade-off in a migratory bird

**DOI:** 10.1186/s12983-016-0179-4

**Published:** 2016-10-10

**Authors:** Patrycja Podlaszczuk, Maciej Kamiński, Radosław Włodarczyk, Krzysztof Kaczmarek, Tomasz Janiszewski, Piotr Minias

**Affiliations:** 1 Department of Biodiversity Studies and Bioeducation, University of Łódź, Banacha 1/3, 90-237 Łódź, Poland; 2 Department of Ecology and Vertebrate Zoology, University of Łódź, Banacha 1/3, 90-237 Łódź, Poland; 3 Medical University of Łódź, Sterlinga 1/3, 91-425 Łódź, Poland

**Keywords:** Common snipe, Feather quality, Gallinago gallinago, Haemoglobin concentration, Life-history, Moult-migration overlap

## Abstract

**Background:**

Moult is one of the most costly activities in the annual cycle of birds and most avian species separate moult from other energy-demanding activities, such as migration. To this end, young birds tend to undergo the first post-juvenile moult before the onset of migration, but in some species the time window for the pre-migratory feather replacement is too narrow. We hypothesized that in such species an increased investment in the structural quality of juvenile feathers may allow to retain juvenile plumage throughout the entire migratory period and delay moult until arriving at wintering grounds, thus avoiding a moult-migration overlap.

**Methods:**

The effect of juvenile plumage quality on the occurrence of moult-migration overlap was studied in a migratory shorebird, the common snipe *Gallinago gallinago*. Ca. 400 of first-year common snipe were captured during their final stage of autumn migration through Central Europe. The quality of juvenile feathers was assessed as the mass-length residuals of retained juvenile rectrices. Condition of migrating birds was assessed with the mass of accumulated fat reserves and whole-blood hemoglobin concentration. Path analysis was used to disentangle complex interrelationships between plumage quality, moult and body condition.

**Results:**

Snipe which grew higher-quality feathers in the pre-fledging period were less likely to initiate moult during migration. Individuals moulting during migration had lower fat loads and hemoglobin concentrations compared to non-moulting birds, suggesting a trade-off in resource allocation, where energetic costs of moult reduced both energy reserves available for migration and resources available for maintenance of high oxygen capacity of blood.

**Conclusions:**

The results of this study indicate that a major life-history trade-off in a migratory bird may be mediated by the quality of juvenile plumage. This is consistent with a silver spoon effect, where early-life investment in feather quality affects future performance of birds during migration period. Our results strongly suggest that the juvenile plumage, although retained for a relatively short period of time, may have profound consequences for individuals’ fitness.

**Electronic supplementary material:**

The online version of this article (doi:10.1186/s12983-016-0179-4) contains supplementary material, which is available to authorized users.

## Background

Most biological systems have sequential nature, as the variation in access to resources or variation in the manner in which they are allocated at one point of life usually has future consequences for performance [1, 2]. A mechanism whereby these future asymmetries in performance are driven by early-life processes is known as a ‘silver spoon’ effect [3]. According to the silver spoon hypothesis an individual who develops under favourable circumstances should enjoy a fitness advantage later in life. While there is abundant empirical evidence for silver spoon effects mediated by nestling condition [4–6], information on how an investment in the quality of the first post-natal (juvenile) plumage affects future performance in birds is almost lacking [7].

In birds, development of plumage is one of the most important physiological processes during the pre-fledging stage [8]. The juvenile plumage is preceded by the nestling down and in precocial species it develops during the first weeks of life while still under parental care. Juvenile feathers are simpler in structure (fewer barbs per unit length) and generally weaker than later generations, as chicks usually face a trade-off between investment in feather quality and rapid body growth [9]. It might be expected that the quality of juvenile feathers will have most notable fitness consequences for species in which individuals retain juvenile plumage for the first autumn migration. Higher structural quality of feathers is likely to increase flight performance and thermoregulatory capabilities [10, 11], which may be critical for a successful completion of a long-distance migration. Also, high-quality juvenile feathers should be more wear-resistant [12], which means that birds could retain them longer before they need to be replaced with a next-generation plumage during the post-juvenile moult.

The moult is one of the most costly activities in the annual cycle of birds. Production of feathers requires large amounts of energy and proteins, also substantially increasing basal metabolic rate of a moulting bird [13]. Thus, most avian species separate moult from other energy-demanding activities, such as migration or reproduction [14, 15]. However, several species of birds have been reported to show a moult-migration overlap to a varying degree [14–17], despite the fact that allocation of energy to moult is expected to reduce energy available for migration, causing a serious life-history trade-off. While most passerines undergo post-juvenile moult before the onset of migration [18], many non-passerine species have too narrow time window for the pre-migratory feather replacement. We hypothesized that in such species an increased investment in the structural quality of juvenile feathers may allow to retain juvenile plumage throughout the entire migratory period and delay moult until arriving at wintering grounds, thus, avoiding a moult-migration overlap.

The aim of this study was to investigate a relationship between the quality of juvenile plumage and the occurrence of moult-migration overlap in a migratory shorebird, the common snipe *Gallinago gallinago*. The common snipe breeds in low Arctic and boreal zones throughout entire Palaearctic, and migrates for wintering grounds in South-Western Europe. First-year common snipe show considerable variation in the timing of post-juvenile moult, as some individuals initiate moult during migration while the others delay it until arrival at wintering grounds [19]. To determine the occurrence of moult-migration overlap we quantified moult status in ca. 400 first-year common snipe captured at their final stage of autumn migration through Central Europe. We used a path analysis to test the following hypotheses: 1) high structural quality of juvenile feathers measured as a mass-length residuals should reduce probability of moult initiation during migration; 2) moulting should reduce energy resources available for migration and decrease investment in migration-related physiological processes. To test the latter hypothesis we measured fat reserves, as a primary fuel for migration, and total blood hemoglobin concentration, which determines ability of an organism to effectively satisfy elevated oxygen requirements of the flight muscles during migration.

## Methods

### General field procedures

Common snipe were captured during autumn migration (28 July-24 September) through Jeziorsko reservoir (51°40′N, 18°40′E), central Poland, in 2008–2015. Although the common snipe breeds in Poland and neighbouring Central European countries, we have no evidence that local individuals use Jeziorsko reservoir as a fuelling site prior to autumn migration and ringing recoveries indicate that long-distance migrants from Central Russia are mostly captured at this site [20]. In total, 1271 first-year snipe were caught in mist-nests and walk-in traps during the study period. All birds were ringed and aged by plumage. A set of basic biometrical measurements were collected upon ringing and tarsus length measured with callipers (±0.1 mm) was used as an index of body size. Sex was determined using either molecular methods [21] or discriminant equations developed for the same migratory population of the common snipe [22]. Based on a detailed plumage examination (body and wing coverts, tertials, and rectrices), all birds were classified as non-moulting (in full juvenile plumage) or undergoing the post-juvenile moult. Very few young birds (if any) finish their post-juvenile moult and attain full adult plumage before reaching wintering grounds. In 2008–2015, we captured only 61 snipe in full fresh (recently moulted) adult-like plumage. These birds were mostly likely adults, which typically start a complete post-breeding moult already at the breeding grounds and attain full fresh plumage before migration is finished. At our study site, 68.5 % of migrating snipes that were classified as adults (*n =* 289) had their moult completed, but retained single unmoulted wing coverts, which constituted the only plumage trait allowing age identification. Taking all these into account, all individuals in full fresh adult-like plumage were excluded from further study procedures.

### Feather quality measurements

We chose outermost rectrices for the measurements of feather quality for the following reasons: 1) structural quality of juvenile tail feathers in birds was reported to well correlate with the quality of other contour feathers and, thus, it may be used as a reliable proxy for the quality of entire juvenile plumage [23]; 2) tail feathers are subject to lower mechanical strain than wing feathers and are likely to show smaller abrasion [24]; we observed no visible signs of wear at the tips of juvenile outermost rectrices in snipe captured at our study site; 3) juvenile outermost rectrices are retained throughout most of the post-juvenile moult and are usually shed last in the moult sequence [25]. Consistently with the last prediction, 91.1 % of moulting young snipe that were captured at our study site (*n =* 526) had both outermost juvenile rectrices retained. Adult-like outermost rectrices are easily distinguished from juvenile ones by shape and size (adult feathers are longer and wider with a characteristic incision in the inner web [26]), which allowed to avoid measurements of feathers that were moulted or accidentally replaced before post-juvenile moult. During eight study years, both outermost juvenile rectrices were plucked from 404 first-year birds (moulting and non-moulting). The total length of each feather (the distance from the calamus base to the distal feather tip) was measured twice with digital callipers (± 0.01 mm) and averaged. Repeatability of the measurement calculated as an intra-class correlation coefficient [27] was 0.999. To reduce variability, all feathers were measured by one of the authors (PP). After feathers were dried, their mass was recorded with digital balance to the nearest 0.1 mg. Structural quality of feathers was assessed with residuals of feather mass against length (*F*_1,402_ = 1318.6, *P <* 0.001; *R* ^2^ = 0.77), which provides a size-independent measure of the structural complexity of feathers. It has been showed that positive residuals indicate a wider rachis and a greater density of barbs than negative residuals [28] and, consequently, they are likely to reflect such properties of feathers as higher bending stiffness and resistance to wear [12, 29].

### Condition indices

The size of visible subcutaneous fat depots in the furcular and axillary region was assessed according to a special scale (fat scores from 0 to 4) developed for shorebirds [30]. To express the total size of fat reserves accumulated by each captured snipe, we averaged furculum and axilla fat scores and subsequently transformed the mean value into the units of mass following the formula developed by Minias et al. [19]. A combination of furcular and axillary fat scores has been shown to linearly reflect an increment in fat mass in shorebirds [31]. We have decided to use fat load instead of size-corrected body mass as an index of condition for two reasons. First, fat load directly measures individual energy reserves available for the processes of moult and migration. Second, lean body mass of birds changes considerably during moult via at least two mechanisms: 1) dry body mass increases during moult due to intensive synthesis of proteins, which are temporarily stored in the organism before they are deposited in feathers [32]; 2) water content of the body (mainly observed in skin, feathers, pectoral muscles, and visceral organs) rises considerably during moult [33, 34]. In fact, an increase in fat-free body mass accompanied with reduction in energy stores has been reported for moulting common snipe, indicating that using body mass to compare condition of moulting and non-moulting snipe might by inappropriate [19].

Total blood haemoglobin concentration is known to reliably reflect the potential of an avian organism to satisfy its oxygen demands [35]. In birds, haemoglobin concentration has been reported to correlate with size-corrected body mass [36, 37], diet quality [38, 39], parasitic rates [40, 41], and survival [42], giving a strong support for reliability of this parameter as a measure of individual condition (reviewed in [43]). For the purpose of the measurement we collected approx. 5 μl of blood from the ulnar vein of each snipe (*n =* 404). Total blood hemoglobin concentration was determined using a portable HemoCue Hb 201+ photometer (HemoCue Hb, Ängelholm, Sweden). HemoCue photometer is acknowledged to reliably measure haemoglobin concentration in avian blood [44] and it is widely used in field and experimental studies on birds [39, 45]. In a subsample of 224 individuals we also measured haematocrit (the packed cell volume), another frequently used indicator of condition in wild birds (e.g. [46, 47]). For this purpose, 40 μl of blood was taken into a heparinized capillary tube and centrifuged at 6000 rpm for 5 min within an hour of collection. Although total blood haemoglobin concentration and haematocrit are based on different biological principles (blood biochemistry and blood cytology, respectively) and may show different sensitivity to ecological factors [48], both parameters well correlated in the common snipe (*r =* 0.52, *P <* 0.001), further supporting reliability of blood haemoglobin concentration to indicate condition in this species.

### (d) Statistical analyses

**Fig. 1:**
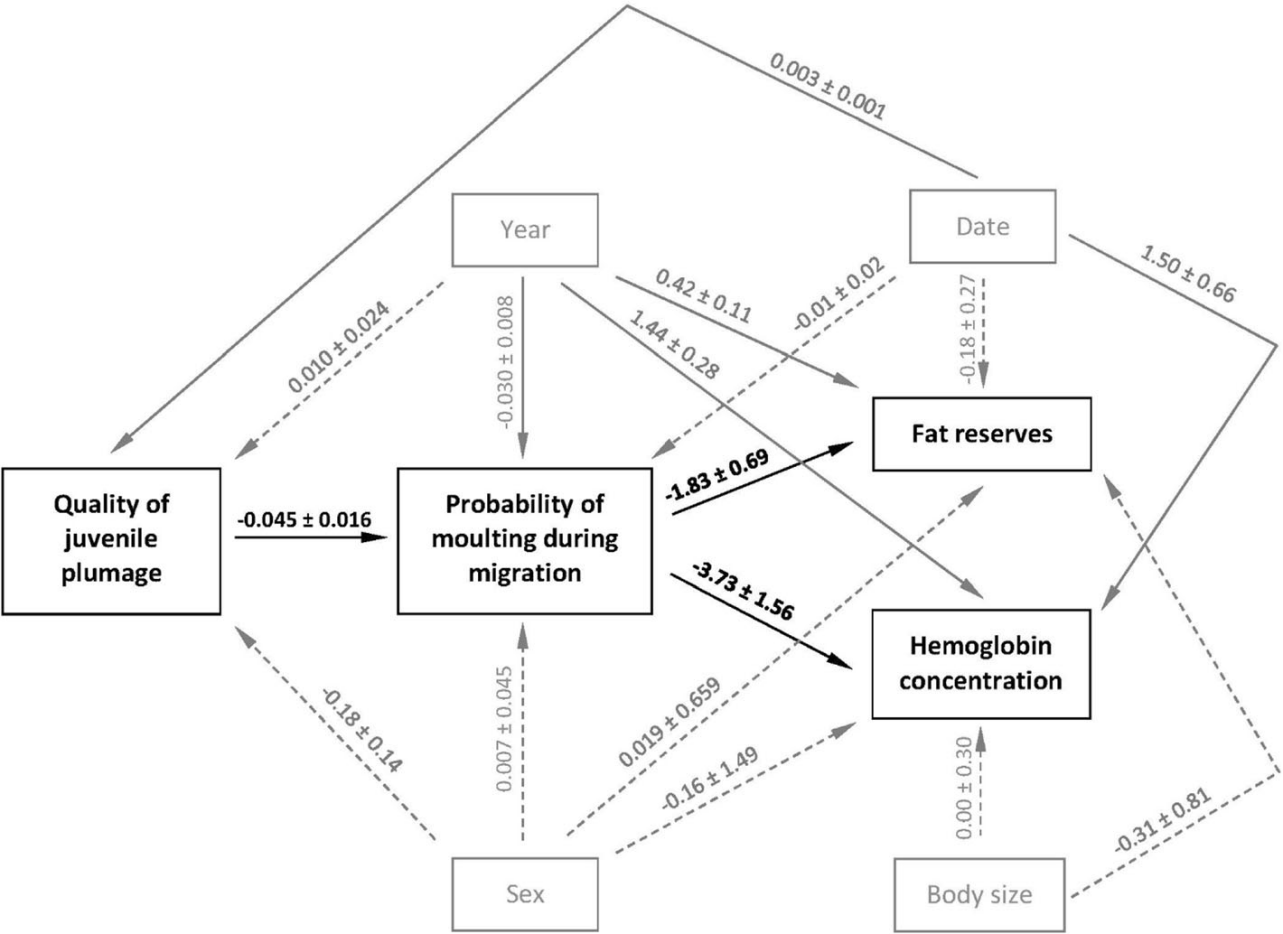
Path diagram showing the relationships between the quality of juvenile feathers, moulting and condition of migrating common snipe. The effects of confounding variables (year, date, sex, and body size) are shown in *grey*. Solid *arrows* represent the significant paths. Coefficient estimates ± SE are presented for each path

Because of complex interrelationships between the main variables (quality of juvenile plumage, probability of moulting, fat reserves, and blood haemoglobin concentration) and confounding effects, we used a path analysis which allows to model directed dependencies among a set of variables. The following confounding variables were included in the analysis: year, date of capture, sex, and body size (expressed with tarsus length). Date of capture was entered to account for the effect of inter-population variation in the analysed traits, as there likely is a temporal shift in the populations of the common snipe that migrate through Central Europe [20]. By including this effect we also controlled for intra-seasonal changes in fat reserves and blood haemoglobin concentration. The effect of sex was entered to control for the possible differences in migration and moult strategies of male and female snipe. It also controlled for the possible variation in oxygen transportation capacity of blood mediated by the differences in endocrinology and hormones between males and females [49]. Body size was entered as the confounding predictor of fat reserves and haemoglobin concentration, because both traits are known to depend on the structural size of birds [50, 51]. Path analysis was conducted in Statistica 10.0 (StatSoft, Tulsa, USA). As data departured from multivariate normality and relatively large sample size was available for the analysis, we used an asymptotically distribution free discrepancy function [52], which followed other studies in ecology (e.g. [53]). All non-significant confounding variables were retained in the model (Fig. 1). All values are reported as means ± SE.

## Results

**Fig. 2:**
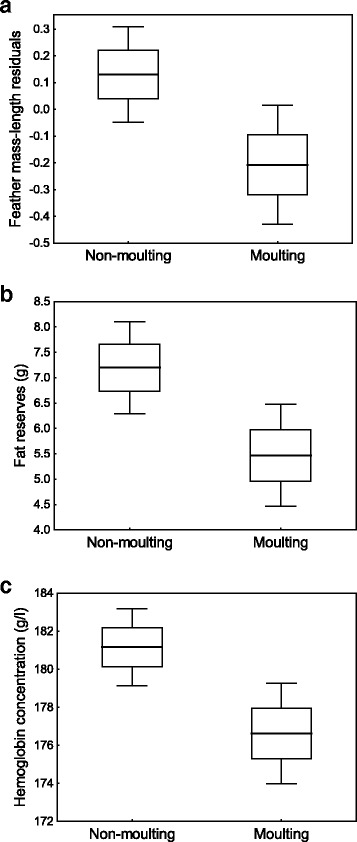
Structural quality of juvenile plumage measured with mass-length residuals of juvenile tail feathers (**a**), fat reserves (**b**) and total blood hemoglobin concentration (**c**) in relation to post-juvenile moult in migrating common snipe. *Horizontal line* – mean, box –SE, whiskers – 95 % confidence intervals

We found that 38.6 % of young snipe (*n =* 404) migrating through our study site had initiated post-juvenile moult and probability of moulting did not vary with sex (*T =* 0.16, *P =* 0.88) (Fig. 1). The effect of date on moult initiation approached significance (*T =* 1.86, *P =* 0.063), suggesting that late migrants were more likely to be in active moult when compared to early migrants (Fig. 1). After controlling for the effect of year (*T =* 3.92, *P <* 0.001), we found that probability of moult initiation during migration was significantly affected by the quality of juvenile plumage (*T =* 2.84, *P =* 0.004; Fig. 1), as snipe with higher mass/length residuals of juvenile feathers had lower probability of post-juvenile moulting (estimate: −0.045 ± 0.016; Fig. 2a). The process of moult negatively affected condition of migrating birds, as moulting snipe had significantly lower amounts of fat reserves (*T =* 2.67, *P =* 0.008; estimate:−1.83 ± 0.69; Fig. 2b) and whole-blood hemoglobin concentration (*T =* 2.39, *P =* 0.017; estimate:−3.73 ± 1.56; Fig. 2c). Both fat reserves and blood hemoglobin concentration varied with date (*T =* 3.43, *P =* 0.001; *T =* 2.94, *P =* 0.003, respectively), indicating that condition of birds increased over the course of migratory season (Fig. 1). By contrast, neither of condition indices was affected by sex (*T =* 0.03, *P =* 0.98; *T =* 0.10, *P =* 0.92, respectively) or body size (*T =* 0.001, *P =* 0.99; *T =* 0.38, *P =* 0.71, respectively). Similarly, we found no evidence for between-sex differences in the structural quality of juvenile feathers (*T =* 1.35, *P =* 0.18).

## Discussion and conclusions

The results of this study provided evidence for a link between structural quality of juvenile feathers of the common snipe and the probability of initiating post-juvenile moult during the first autumn migration. We found that snipe which grew higher-quality feathers in the pre-fledging period were more likely to retain them until arrival at wintering grounds, thus avoiding a moult-migration overlap. Also, individuals moulting during migration showed substantial trade-off in resource allocation, as energetic costs of moult significantly reduced both fat reserves available for migration and resources available for maintenance of high oxygen capacity of blood (measured with blood haemoglobin concentration).

Our findings are generally consistent with a silver spoon effect, where early-life investment in feather quality affected future performance of birds during migration period. While there is ample experimental evidence for the effects of nestling condition on plumage colouration in birds [54–56], studies that link rearing conditions to structural quality of juvenile feathers are lacking. Nevertheless, it has been recognized that the amount of resources invested in feathers is likely to show high phenotypic plasticity within individuals [57]. For example, feather mass of the pied flycatcher *Ficedula hypoleuca* has been reported to be largely determined by environmental effects experienced during feather growth [57]. Thus, we think it is reasonable to assume that non-negligible proportion of variance in the structural quality of snipe juvenile feathers is likely explained with conditions experienced during the natal period, when feathers are formed. While this scenario supports silver spoon hypothesis, we also acknowledge that feather quality may have a relatively high genetic component. Heritability (*h* ^2^) for feather mass in the pied flycatcher has been estimated to range between 0.59–0.65 [57] and we agree that both environmental and genetic factors could shape variation in feather quality that we observed in migrating common snipe.

Silver spoon effects mediated by post-natal plumage quality have been surprisingly rarely documented in avian studies. As far as we are aware, the only evidence for this mechanism comes from greenfinch *Carduelis chloris*, in which high structural quality and carotenoid chroma of juvenile feathers were positively linked to the extent of post-juvenile moult, allowing young birds to attain more adult-like plumage during first autumn [7]. Greenfinches with high-quality juvenile plumage were also in better condition during the first winter period, although it is uncertain whether this was due to thermoregulatory or flight-related benefits of juvenile feathers *per se* or due to the higher plumage-dependent social status of birds [7].

While favourable rearing conditions may be beneficial throughout life [58–60], they are likely to be most pronounced during early life stages, as potential long-term silver spoon effects are limited by accumulating environmental stochasticity that individuals experience during life [61]. Consistently, most evidence for the silver spoon effect in birds comes from short-term associations, where rearing conditions affect different components (e.g. survival, dispersal) of post-fledging performance [5, 62–64]. It seems surprising though, that information on how rearing conditions affect migration performance is almost lacking. Rare evidence for carry-over effects of early-life events on migratory performance has been presented for the Savannah sparrow *Passerculus sandvichensis* [65]. It was shown that nestling condition of sparrows positively correlated with fat stores accumulated immediately prior to migration and negatively correlated with fledging date. Conditions during development also limited survival during migration through their effect on fat stores [65]. Similarly, nestling body condition was found to be a good predictor of amount of subcutaneous fat during migration in blue tits *Cyanistes caeruleus* [66]. Several different mechanisms have been proposed to explain these correlations, among the others: 1) higher susceptibility to disease in individuals reared under poor conditions, resulting in additional maintenance costs that limit fat accumulation [67]; 2) impaired cognitive function of individuals that developed in nutritional stress, which may reduce foraging efficiency [68]; and 3) exclusion of individuals that fledge in poor condition from favourable foraging sites [69]. We suggest that the correlation between poor nestling body condition and migratory performance may be also mediated by the quality of post-natal plumage, as individuals raised under unfavourable conditions are likely to grow low-quality plumage [70], ensuring worse (more energetically expensive) flight performance and impaired thermoregulation [11].

While migratory performance could be affected by plumage quality *per se*, in the common snipe it has been probably mediated by the effect of plumage quality on the timing of moult initiation. Feathers of low structural quality are less resistant to wear and abrasion [12] and, thus, it seems likely that individuals with low quality plumage developed in the post-natal period may not be able to complete their first autumn migration without, at least partial, feather renewal. In turn, initiation of moult during migration may entail serious consequences for migratory performance. Thermoregulatory costs and basal metabolic rate increase considerably during the time of moulting [71, 72]. Also, feather synthesis and other moult-related physiological processes require huge energy expenditure [13, 73], which may highly limit resources available for migration. Such physiological trade-off between moult and migration has been reported for several avian species, as moulting was shown to impair pre-migratory fat accumulation [74, 75] or to provoke faster depletion of fat reserves during migration [14]. Additionally, moulting during migration could produce a poor-quality plumage, as birds overlapping moult with other energy demanding activities have been shown to grow lighter and shorter feathers [76]. Although our data did not allow to determine whether the observed relationships are of causal or correlative nature, we are convinced that they are unlikely to result from inter-population differences in the timing of migration through our study site (and, consequently, from possible inter-population differences in plumage quality and the timing of moult initiation), as we carefully controlled for the temporal variation in all the analysed traits.

To conclude, we provided the first evidence, although non-experimental, for a serious life-history trade-off mediated by the quality of juvenile plumage in a migratory bird. Our results strongly suggest that the juvenile plumage, although retained for a relatively short period of time, may have profound consequences for individuals’ fitness.

## Additional file

Additional file 1: Raw data for feather measurements, moult status, and migratory traits of the first-year common snipe. (XLSX 28 kb)
